# Monitoring the Growth and Yield of Fruit Vegetables in a Greenhouse Using a Three-Dimensional Scanner

**DOI:** 10.3390/s20185270

**Published:** 2020-09-15

**Authors:** Yuta Ohashi, Yasuhiro Ishigami, Eiji Goto

**Affiliations:** 1 Graduate School of Horticulture, Chiba University, Matsudo, Chiba 271-8510, Japan; y-ohashi@chiba-u.jp; 2 Faculty of Agriculture, Takasaki University of Health and Welfare, Takasaki, Gunma 370-0033, Japan; ishigami@takasaki-u.ac.jp; 3 Plant Molecular Research Center, Chiba University, Chiba 260-0856, Japan

**Keywords:** canopy structure, *Capsicum annuum*, *Cucumis sativus*, dry matter, image analysis, leaf are index, leaf area, plant height, *Solanum lycopersicum*, yield

## Abstract

Monitoring the growth of fruit vegetables is essential for the automation of cultivation management, and harvest. The objective of this study is to demonstrate that the current sensor technology can monitor the growth and yield of fruit vegetables such as tomato, cucumber, and paprika. We estimated leaf area, leaf area index (LAI), and plant height using coordinates of polygon vertices from plant and canopy surface models constructed using a three-dimensional (3D) scanner. A significant correlation was observed between the measured and estimated leaf area, LAI, and plant height (R^2^ > 0.8, except for tomato LAI). The canopy structure of each fruit vegetable was predicted by integrating the estimated leaf area at each height of the canopy surface models. A linear relationship was observed between the measured total leaf area and the total dry weight of each fruit vegetable; thus, the dry weight of the plant can be predicted using the estimated leaf area. The fruit weights of tomato and paprika were estimated using the fruit solid model constructed by the fruit point cloud data extracted using the RGB value. A significant correlation was observed between the measured and estimated fruit weights (tomato: R^2^ = 0.739, paprika: R^2^ = 0.888). Therefore, it was possible to estimate the growth parameters (leaf area, plant height, canopy structure, and yield) of different fruit vegetables non-destructively using a 3D scanner.

## 1. Introduction

It is vital to increase the efficiency of agricultural work because of the high labour cost [1], highlighting the need for automation. Farmers continue to seek methods to increase productivity using small numbers of individuals [2]. In greenhouse horticulture, environmental control (for example, temperature, solar radiation, CO_2_ and vapor-pressure deficit [VPD]) systems have been developed using information and communication technology (ICT) [3]. In the future, there will be a need to automate harvesting and cultivation management in order to save energy. Monitoring the growth of fruit vegetables will provide input data that can be used to control robots for cultivation management and harvesting. Higashide (2018) showed that when the leaf area index (LAI) was increased, the amount of solar radiation in the lower canopy was lower than the light compensation point, meaning that it could not contribute to photosynthesis due to the consumption of assimilation products by respiration [4]. Therefore, appropriate leaf thinning increases the yield of fruit vegetables; hence, the technique used to monitor the growth of fruit vegetables should also consider the timing of leaf thinning. Recently, the growth of trees and grains has been estimated using two- (2D) or three-dimensional (3D) information.

Dornbusch et al. (2007) constructed 3D structural models of barley leaves and stems using triangles calculated by 3D point cloud data to extract morphological traits [5]. The point cloud is a set of data points comprising coordinates in a space. Benalcázar et al. (2011) extracted soybean leaves from a 2D image, including the background, using the hue, saturation, intensity (HSI) color model, and calculated the leaf area using the number of leaf pixels [6]. Casadesús and Villegas (2014) estimated the LAI and dry weight of wheat by calculating the ratio of green pixels from multiple images [7]. Hosoi and Omasa (2006) estimated the leaf area density (LAD) and LAI of trees using solid models constructed by a LIDAR, which precisely reproduced the canopy [8]. In addition, the solid model has been used to predict the volume of trees [9]. Dandois et al. (2015) estimated the canopy height of a deciduous forest using a 3D multispectral point cloud acquired by unmanned aerial vehicle-structure from motion (UAV-SFM) remote sensing [10]. Lati et al. (2013) estimated the plant height, leaf area, and fresh weight of corn and cotton using solid models constructed by 3D stereovision modeling [11]. The use of single-image 2D analysis to estimate growth parameters was affected by imaging position, plant density, and species compared with the use of a 3D analysis [11]. Hosoi et al. (2011) estimated the leaf area and LAI of tomato using the polygon area of a canopy surface model scanned from three places by the LIDAR [12]. Ohashi et al. (2020) estimated the individual leaf area of tomato using the polygon area of plant surface models constructed by a 3D scanner [13]. Itakura and Hosoi (2018) constructed surface models of small plants such as pothos and hydrangea from multiple photos, which were segmented automatically using the watershed algorithm to estimate leaf area and angle of inclination [14]. Zhang et al. (2018) estimated the plant height, leaf number, and leaf area of sweet potato under different fertilizer conditions using surface models constructed by the structure from motion (SfM) method, and observed a linear relationship between measured and estimated value [15]. Itakura and Hosoi (2019) estimated the leaf area and inclination angle of eggplant, pea, and common bean using solid models constructed by the SfM method, and the absolute error was found to be 8.87% [16].

Fruits of fruit vegetables have also been detected using 2D or 3D images in order to automate the harvest. For example, Yamamoto et al. (2014) developed a method that could accurately identify individual tomato fruits, including immature fruits, on a plant using an RGB digital camera with machine learning approaches based on classification models of image color, shape, texture, and size [17]. Zhao et al. (2016) detected ripe tomato fruits based on color analysis with machine learning and achieved 96% detection accuracy [18]. Hashimoto et al. (2012) reported that the change in tomato surface color during maturation could be measured without the influence of solar radiation when color was calibrated between morning and evening [19]. Yaguchi et al. (2018) detected tomato fruits using color information from point cloud data acquired by a 3D camera, and reported that tomato fruits were harvested at 10 s per fruit using a harvesting robot equipped with cutting scissors and rotational plucking gripper [20]. Ohmori et al. (2015) detected tomato fruits based on the tone, value, and hue of a plant image and successfully harvested 76.9% of fruits using a robot [21].

In orchards, fruits have been detected by image analysis. For example, Tu et al. [22] were able to detect passion fruit and classify ripeness using a neural network from RGB-D images, with an accuracy of 92.71 and 91.52%, respectively. Guava fruits were detected using RGB-D images to enable automatic harvesting, without touching branches, using a robot [23]. In addition, the size of mango fruit [24] and the location of apple [25] were estimated using RGB-D images. Fruits have been frequently detected using image analysis. Fully automated estimation of yield before harvesting is important for precision agriculture [17]. Estimating the yield and distribution of fruits provides useful information for staffing and sales planning.

Although studies have monitored the growth of fruit vegetables, few have investigated tall fruit vegetables, except for fruit detection. For example, Rose et al. (2015) estimated the leaf area and plant height of tomato using 3D models; however, small tomato plants were used, 3 weeks after sowing [26]. In addition, few studies have estimated fruit weight based on 2D or 3D images. Presently, growth surveys of fruit vegetables have been conducted using a ruler and by destructive methods; however, these surveys require a lot of energy and time. Therefore, it is important to estimate multiple growth parameters simultaneously and non-destructively in order to optimize cultivation management and automation.

The objective of this study is to demonstrate that the current sensor technology can monitor the growth and yield of fruit vegetables such as tomato, cucumber, and paprika. In this study, point cloud data were acquired by a 3D scanner to simultaneously estimate growth parameters of fruit vegetables, including leaf area, LAI, dry weight, plant height, canopy structure, and fruit weight, in a non-destructive manner.

## 2. Materials and Methods

### 2.1. Test Plants and Greenhouse

**Figure 1:**
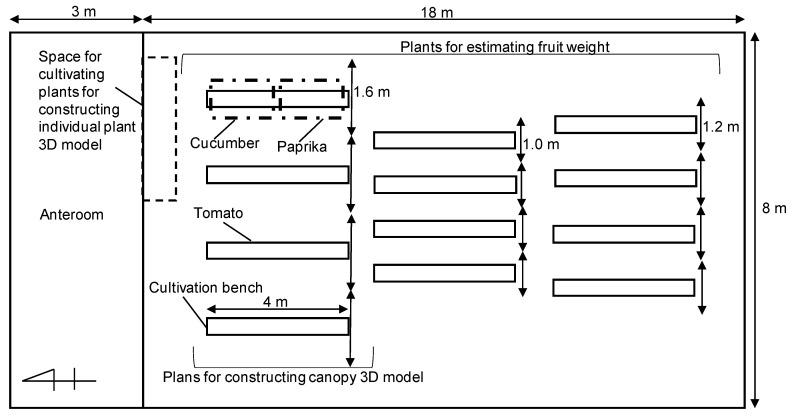
Location of cultivation benches and plants (tomato, cucumber, and paprika) in the greenhouse. The greenhouse was located in Matsudo, Chiba, Japan.

Three-week-old tomato (*Solanum lycopersicum* L., ‘Reika’, Sakata Seed Co., Ltd., Yokohama, Japan), cucumber (*Cucumis sativus* L., ‘Freedom-house No. 2′, Sakata Seed Co., Ltd., Kyoto, Japan), and paprika (*Capsicum annuum* L., ‘Frupi-yellow’, TAKII Co., Ltd., Kyoto, Japan) were transplanted to rockwool cubes (DELTA6.5G, Grodan Inc., Roermond, Netherlands) on rockwool slabs (2075 A2W, Grodan Inc., Roermond, The Netherlands) on 21 March 2019 in a north-south greenhouse (length: 21 m, width: 8 m, average height: 4 m, area: 168 m^2^), and covered by a polyolefin film. The greenhouse was located in Matsudo, Chiba, Japan. Plants were grown on cultivation benches in the greenhouse (Figure 1) and arranged in a zigzag pattern, with 33 cm between plants. The canopies were used to estimate the LAI, canopy structure, and fruit weight.

Three-week-old tomato, cucumber, and paprika were cultivated using deep flow technique (DFT) from 21 March to 2 April 2019, after which they were transplanted to a wagner pot (AS ONE Co., Ltd., Osaka, Japan) filled with rock wool (Granulate, Grodan Inc., Roermond, Netherlands). The above plants were used to estimate leaf area, dry weight, and plant height.

Air temperature and vapor pressure inside the greenhouse were measured and controlled by an integrated environmental control system (Profarm-controller, Denso Co., Ltd., Kariya, Japan). Photosynthetic photon flux density (PPFD) inside the greenhouse was measured by a PPFD sensor (ML-020P sensor, EKO Instruments Co., Ltd., Tokyo, Japan). The average air temperature, vapor pressure, and daily integrated PPFD during the cultivation period (21 March–5 September) inside the greenhouse were 23.6 °C, 0.59 kPa, and 15.6 mol m^−2^ d^−1^, respectively. A one-fold concentration of A recipe (OAT Agrio Co., Ltd., Tokyo, Japan) was used as a nutrient solution.

### 2.2. Growth Monitoring

#### 2.2.1. Obtaining Point Cloud Data of Plants

**Figure 2:**
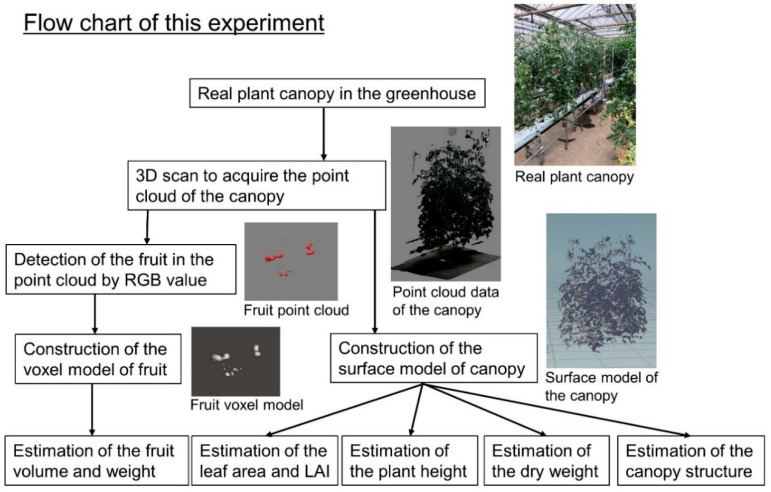
Flow chart of monitoring of the growth and yield of fruit vegetables using a three-dimensional (3D) scanner. LAI, leaf area index.

A flow chart of the study is shown in Figure 2. A 3D scanner (DPI-8X, Opt Technologies Co., Ltd., Tokyo, Japan) scanned around the individual plants at sunset in order to acquire point cloud data for use to estimate leaf area, dry weight, and plant height. The PrimeSense Carmine sensor 1.08 (Apple Inc., Cupertino, CA, USA) was installed onto the 3D scanner. The measuring range was from 0.6 to 4.0 m (spatial x/y resolution at 2 m was 3.4 mm, depth resolution at 2 m was 12 mm). When the measured distance was 1.0, 2.0, and 3.3 m, the accuracy of the 3D scanner was 2, 6, and 10 mm, respectively. When the point cloud data was acquired, the measured distance between the 3D scanner and plant canopy ranged from 0.6 to 1.0 m. Each point cloud comprised XYZ coordinates. Tomatoes were scanned from 7 April to 19 May (17–59 days after transplanting (DAT)), cucumber from 7 April to 19 May (5–47 DAT), and paprika from 7 April to 12 June (5–71 DAT) about once a week at different growth stages (plant height and leaf area). After scanning, a destructive survey was conducted to measure leaf area, plant height, and dry weight (leaf and stem without a fruit) per plant. Leaf area was measured by an area meter (LI-3100, LI-COR Inc., Lincoln, NE, USA).

Canopies composed of six plants were scanned using the 3D scanner at sunset to estimate LAI and canopy structure. Tomatoes were scanned from 5 April to 23 May (15–63 DAT), cucumber from 3 April to 23 May (1–51 DAT), and paprika from 5 April to 2 August (3–122 DAT) at different growth stages. After scanning, leaf length and width were measured. An equation (leaf area = leaf length × leaf width × coefficient) was used to calculate the leaf area of the canopy. The above equation has been applied to various plants [27,28,29]. Leaf area was calculated using the following Equation (1) to calculate the LAI of the canopy in a non-destructive manner.

(1) $$\mathrm{Leaf}\;\mathrm{area}\;\left({\mathrm{cm}}^{2}\right)=a\times \mathrm{leaf}\;\mathrm{length}\;\left(\mathrm{cm}\right)\times \mathrm{leaf}\;\mathrm{width}\;\left(\mathrm{cm}\right)$$

Here, a is a plant-dependent parameter (tomato 0.25, cucumber 0.87, paprika 0.59). We measured the leaf area, leaf length, and leaf width to investigate the relationship between the leaf area and leaf length × leaf width and to calculate a in Equation (1). The values of R^2^ between the two datasets for tomato, cucumber, and paprika were 0.902, 0.981, and 0.980. Color information from the point cloud data was deleted to limit the inclusion of unnecessary information.

#### 2.2.2. Construction of a Surface Model for Growth Estimation

**Figure 3:**
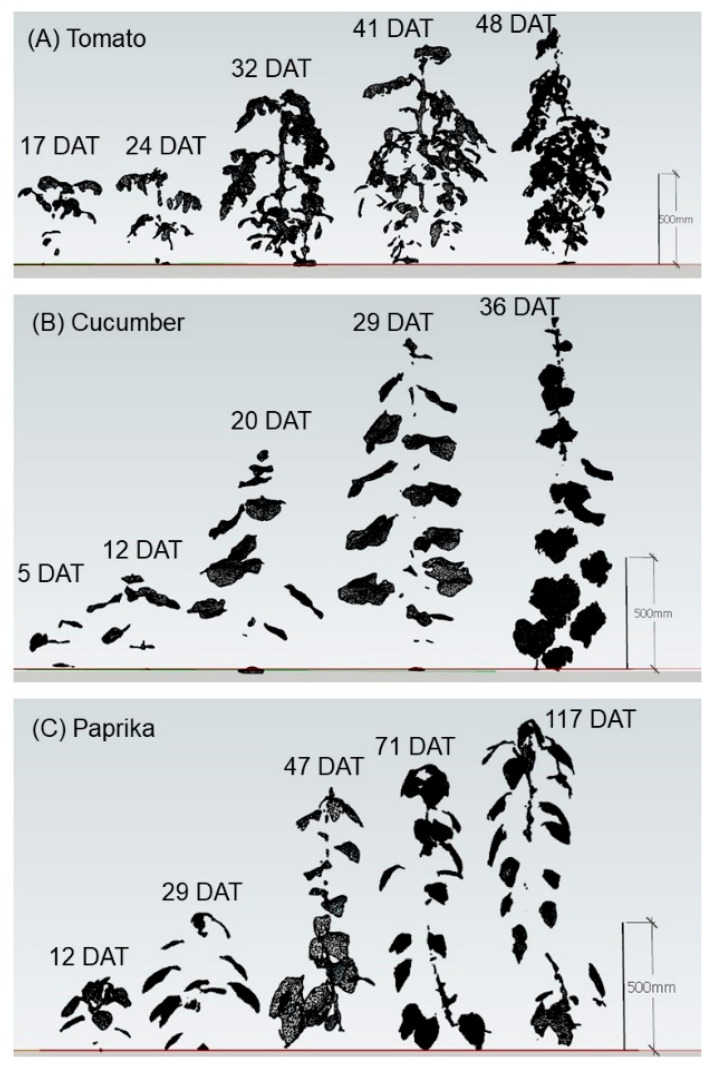
Surface model of individual plants to estimate leaf area, dry weight, and plant height. Tomato plants were scanned using a 3D scanner (DPI-8X, Opt Technologies Co., Ltd., Tokyo, Japan) from 7 April to 19 May 2019 (17–59 days after transplanting (DAT)) (**A**). Cucumber plants were scanned from 7 April to 19 May 2019 (5–47 DAT) (**B**). Paprika plants were scanned from 7 April to 28 July 2019 (5–117 DAT) (**C**). Individual plant surface models were constructed using SketchUp 2017 (Trimble Inc., Sunnyvale, CA, USA).

**Figure 4:**
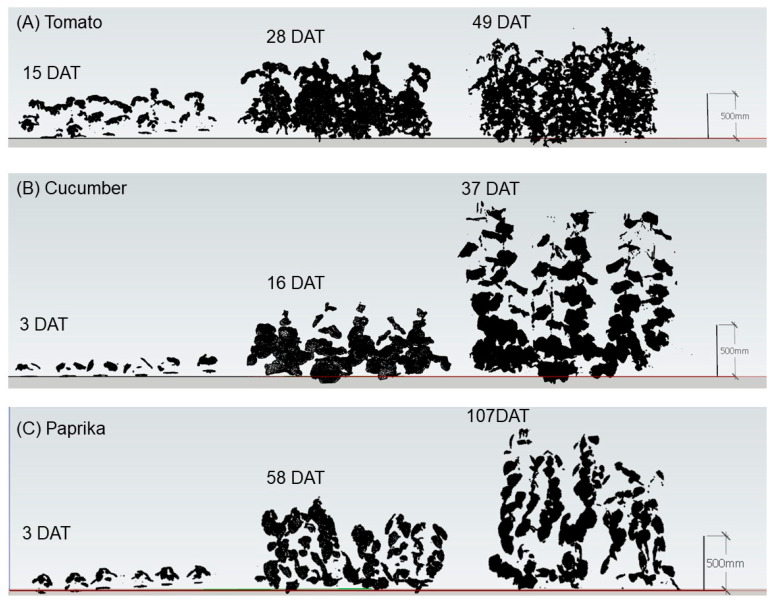
Surface model of plant canopies for estimation of LAI and plant structure. Tomato canopies were scanned using the 3D scanner (DPI-8X, Opt Technologies Co., Ltd., Tokyo, Japan) from 5 April to 23 May 2019 (15–63 days after transplanting (DAT)) (**A**). Cucumber canopies were scanned from 3 April to 23 May 2019 (1–51 DAT) (**B**). Paprika canopies were scanned from 5 April to 2 August 2019 (3–122 DAT) (**C**). Plant canopy surface models were constructed using SketchUp 2017 (Trimble Inc., Sunnyvale, CA, USA). LAI, leaf area index.

The point cloud data for individual plants and canopies (see Section 2.2.1) was converted to a surface model, which was constructed by polygons with three coordinates in standard triangulated language (STL) format using OPT Cloud Survey (Opt Technologies Co., Ltd., Tokyo, Japan). Therefore, it was possible to calculate the surface area of the surface model using the polygon. Unnecessary parts of the surface model were trimmed using Houdini FX 17 (Side Effects Software Inc., Toronto, ON, Canada) (Figure 3 and Figure 4).

The polygon area was calculated from the plant surface model to estimate leaf area as follows.

In order to calculate the surface area of a given polygon ΔOAB, the values of its cross products $\vec{OA}$ and $\vec{OB}$ were halved. If $\vec{OA}$ and $\vec{OB}$ are defined as $\vec{OA}$ = (a_1_, a_2_, a_3_) and $\vec{OB}$ = (b_1_, b_2_, b_3_), respectively, then the following Equation (2) could be used to calculate the surface area of ΔOAB.

(2) $$\Delta \mathrm{OAB}=\frac{1}{2}\times \sqrt{{\left(a_{2}b_{3}-a_{3}b_{2}\right)}^{2}+{\left(a_{3}b_{1}-a_{1}b_{3}\right)}^{2}+{\left(a_{1}b_{2}-a_{2}b_{1}\right)}^{2}}$$

This equation was applied to all polygons of the plant surface model, and the leaf area was estimated to integrate the area of all polygons. We wrote a simple program in Python to read the coordinates of the polygons (up to ~400 thousands) in the STL file and calculated the leaf area. LAI was estimated using the leaf area of canopy calculated by the canopy surface model, and the cultivation area.

The relationship between the measured leaf area and the dry weight of leaf and total (leaf and stem without fruits) was investigated to estimate the dry weight of the plant. If a linear relationship was found between leaf area and dry weight, the dry weight of the plant could be predicted from the estimated leaf area. The relationship between the estimated leaf area and the measured dry weight of leaves and total dry weight was investigated.

Plant height from the base to the growing point was estimated using the measuring tool of SketchUp 2017 (Trimble Inc., Sunnyvale, CA, USA).

The regression analysis and calculation of root mean square error (RMSE) were conducted between the measured and estimated value to obtain their accuracy.

Canopy structure, which is a vertical distribution of leaf area, was estimated using the estimated leaf area at each height.

### 2.3. Yield Monitoring (Tomato and Paprika)

#### 2.3.1. Detection of Fruits from Canopy Point Cloud Data Using RGB Values

The 3D scanner was used at sunset to scan the canopy composed of six plants of tomato from 14 August to 5 September 2019 (146–168 DAT) and six paprika from 28 August to 30 2019 (148–150 DAT) to acquire canopy point cloud data. The plant height, number of leaves, and LAI of tomato were ~150 cm, ~20, and ~3, respectively. The plant height, number of leaves, and LAI of paprika were ~160 cm, ~35, and ~2, respectively. After the tomato canopy with 3–23 mature fruits was scanned, the total fresh weight of mature fruits inside the canopy was measured. The above measurements were made several times during the tomato cultivation period. A dataset was created including the tomato canopy point cloud and the weight of mature fruits. After the paprika canopy was scanned and the two mature fruits were harvested, the fresh weight of those fruits was measured. The above measurements were repeated until all fruits were harvested. A dataset was created including the paprika canopy point cloud and the weight of mature fruits. The method used to measure fruit weight differed between tomato and paprika because the time required for paprika fruit to ripen is longer than that for tomato, and it was difficult to prepare a variety of paprika canopies.

The point cloud data acquired in this study included plants and fruits. It was possible to differentiate fruits from color images of the canopy [30]. The point cloud acquired by the 3D scanner had XYZ coordinates and RGB values. The RGB value is a value of 0–255 that is used to express red, green, and blue [31]. The point cloud data were outputted in PTS format using OPT Cloud Survey. In the experiment described in Section 2.2.2, the RGB value of point cloud data was erased to reduce file size. Conversely, here, RGB values were used to detect fruits.

**Figure 5:**
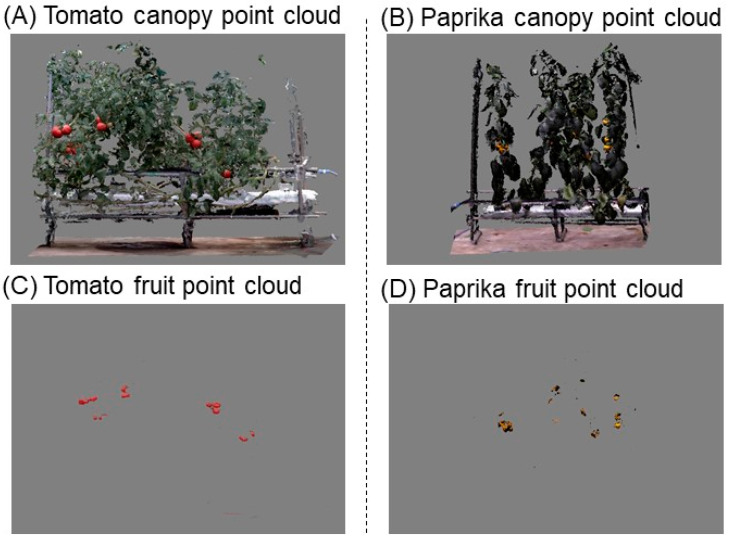
Plant canopy and fruit point cloud data. Tomato canopies for estimation of fruit weight were scanned using the 3D scanner (DPI-8X, Opt Technologies Co., Ltd., Tokyo, Japan) from 14 August to 5 September 2019 (**A**). Paprika canopies for estimating fruit weight were scanned from 28 August to 30 2019 (**B**). Fruit point cloud data were detected from the canopy point cloud data using the RGB value. Point cloud data of tomato fruits fulfilling R > 140, G < 100, and B < 100 were acquired (**C**). Point cloud data of paprika fruits fulfilling R > 150, G > 110, and B < 50 were obtained (**D**). Point cloud data were obtained using OPT Cloud Survey (Opt Technologies Co., Ltd., Tokyo, Japan).

The point cloud data of tomato and paprika fruits fulfilling R > 140, G < 100, B < 100, and R > 150, G > 110, and B < 50 were obtained from the canopy point cloud data (Figure 5). When the above conditions were determined, a color chart was used to identify suitable conditions for detecting tomato and paprika fruits.

#### 2.3.2. Construction of a Solid Model for Estimation of Fruit Weight

**Figure 6:**
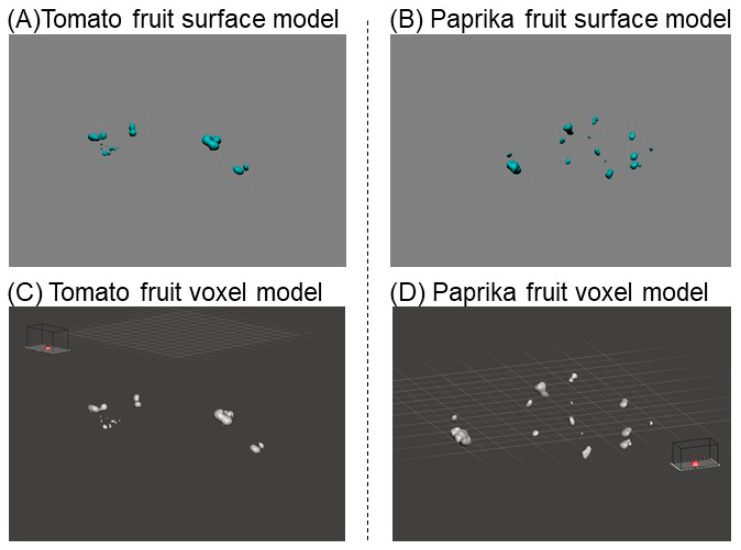
Fruit surface and voxel models. The fruit surface model was constructed by the fruit point cloud using OPT cloud survey (DPI-8X, Opt Technologies Co., Ltd., Tokyo, Japan) (**A**,**B**). The fruit voxel model was constructed from the surface model using Meshmixer 3.5 (Autodesk Inc., San Rafael, CA, USA) (**C**,**D**).

The point cloud data for tomato and paprika fruits (see Section 2.3.1) were converted to a surface model using OPT Cloud Survey (Figure 6A,B). The surface model of fruits was converted to a solid model using Meshmixer 3.5 (Autodesk Inc., San Rafael, CA, USA) (Figure 6C,D). The solid model is full of voxels; therefore, it was possible to calculate volume by counting several voxels [32], whereby a voxel is a cube used to construct a 3D model. The volume of the fruit solid model was calculated by Meshmixer 3.5. The fruit weight was estimated by multiplying the fruit volume (cm^3^) by the density (g cm^−3^). Fruit volume was measured by the water displacement method based on the Archimedes principle [33]. We prepared a plastic beaker poured with water. The fruit was submerged in the beaker. Then, the increased volume was the volume of the fruit. The fruit density was calculated using the volume and weight. The density of tomato and paprika fruit was 0.84 and 0.59 g cm^−3^ (average of five fruits), respectively. The relationship between the measured and estimated fruit weight was investigated. The regression analysis and calculation of RMSE were conducted between the measured and estimated values to obtain their accuracy.

## 3. Results

### 3.1. Growth

**Figure 7:**
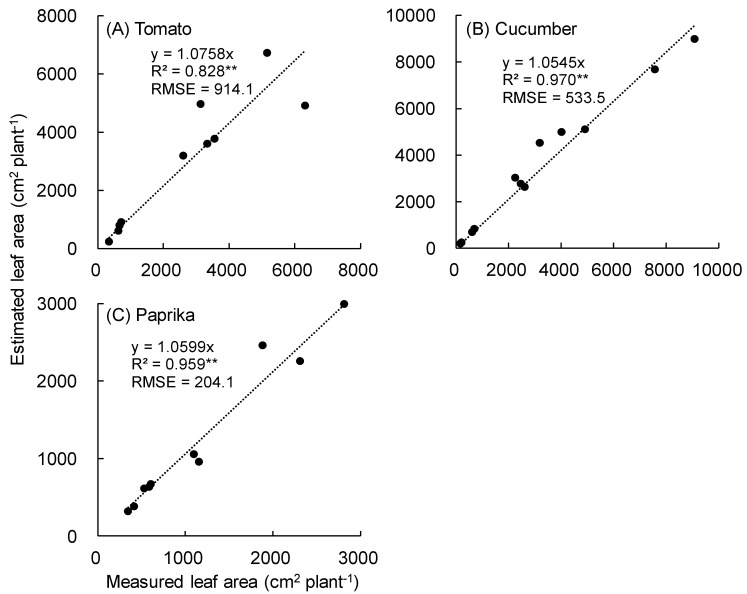
Relationship between measured and estimated leaf area per plant. The leaf area was measured using an area meter (LI-3100, LI-COR Inc., Lincoln, NE, USA). The leaf area was estimated based on the area of polygons in the plant surface model. Tomato plants were scanned using a 3D scanner (DPI-8X, Opt Technologies Co., Ltd., Tokyo, Japan) from 5 April to 23 May 2019 (**A**). Cucumber plants were scanned from 3 April to 23 May 2019 (**B**). Paprika plants were scanned from 5 April to 2 August 2019 (**C**). ** significant at *p* < 0.01 according to the regression analysis. RMSE means root mean squared error.

The relationship between measured and estimated leaf area per plant is shown in Figure 7. Unnecessary polygons were observed as a noise in the tomato surface model. A significant correlation was observed between the measured and estimated leaf area of tomato (R^2^ = 0.828 and RMSE = 914.1 cm^2^) (Figure 7A), cucumber (R^2^ = 0.970 and RMSE = 533.5 cm^2^) (Figure 7B), and paprika (R^2^ = 0.959 and RMSE = 204.1 cm^2^) (Figure 7C).

**Figure 8:**
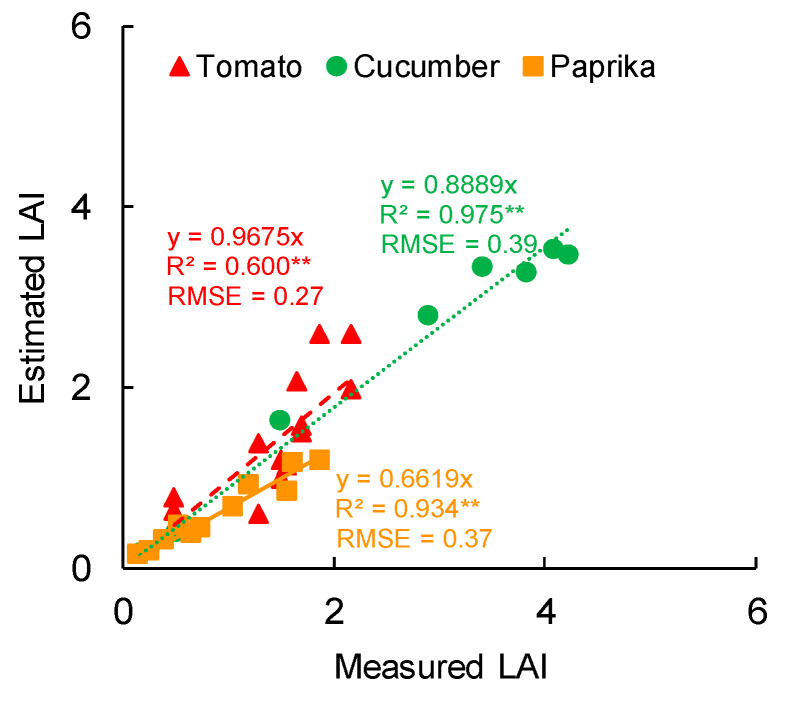
Relationship between measured and estimated leaf area index (LAI). LAI was measured non-destructively using the leaf area (leaf area = leaf length × leaf width × coefficient) and cultivation area. LAI was estimated based on the polygon area of the canopy surface model and cultivation area. Tomato and cucumber canopies were scanned using the 3D scanner (DPI-8X, Opt Technologies Co., Ltd., Tokyo, Japan) from 7 April to 23 May 2019. Paprika canopy was scanned from 7 April to 12 June 2019. ** significant at *p* < 0.01 according to the regression analysis. LAI, leaf area index. RMSE means root mean squared error.

The relationship between measured and estimated LAI is shown in Figure 8. A significant correlation was observed between the measured and estimated LAI of tomato (R^2^ = 0.600 and RMSE = 0.27), cucumber (R^2^ = 0.975 and RMSE = 0.39), and paprika (R^2^ = 0.934 and RMSE = 0.37). The LAI of paprika was underestimated compared with those of tomato and cucumber.

**Figure 9:**
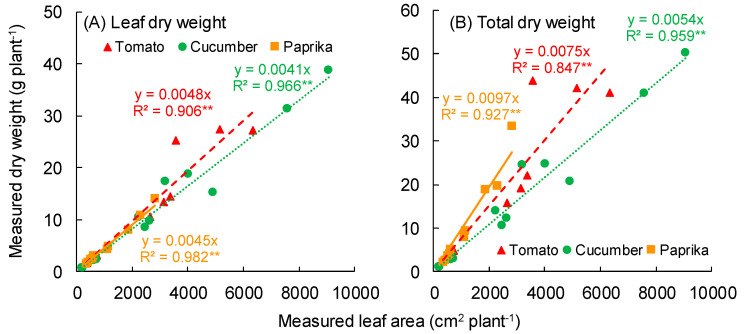
Relationship between measured leaf area and dry weight (leaf or total). (**A**) and (**B**) show the dry weights of leaf and total, respectively. The total dry weight does not include fruit. The leaf area was measured using an area meter (LI-3100, LI-COR Inc., Lincoln, NE, USA). Tomato plants were scanned using the 3D scanner (DPI-8X, Opt Technologies Co., Ltd., Tokyo, Japan) from 5 April to 23 May 2019. Cucumber plants were scanned from 3 April to 23 May 2019. Paprika plants were scanned from 5 April to 2 August 2019. ** significant at *p* < 0.01 according to the regression analysis.

The relationship between measured leaf area and leaf dry weight is shown in Figure 9A. A significant correlation was observed between the measured leaf area and leaf dry weight of tomato (R^2^ = 0.906), cucumber (R^2^ = 0.966), and paprika (R^2^ = 0.982). The relationship between measured leaf area and total dry weight without fruit is shown in Figure 9B. A significant correlation was observed between the measured leaf area and total dry weight of tomato (R^2^ = 0.847), cucumber (R^2^ = 0.959), and paprika (R^2^ = 0.927).

**Figure 10:**
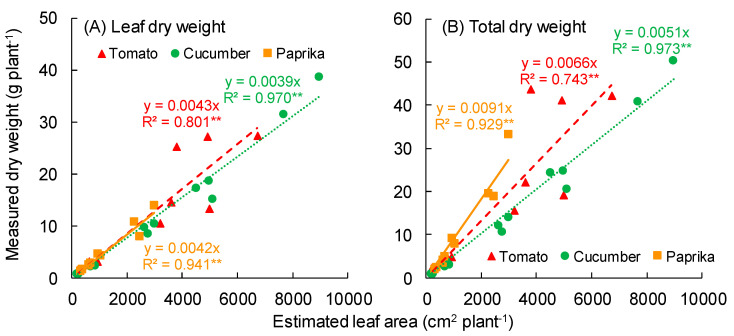
Relationship between estimated leaf area and measured dry weight (leaf or total). (**A**,**B**) show the dry weights of leaf and total, respectively. The total dry weight does not include fruit. The estimated leaf area is the area of polygons in the plant surface model. Tomato plants were scanned using the 3D scanner (DPI-8X, Opt Technologies Co., Ltd., Tokyo, Japan) from 5 April to 23 May 2019. Cucumber plants were scanned from 3 April to 23 May 2019. Paprika plants were scanned from 5 April to 2 August 2019. ** significant at *p* < 0.01 according to the regression analysis.

The relationship between estimated leaf area and measured leaf dry weight is shown in Figure 10A. A significant correlation was observed between the estimated leaf area and measured leaf dry weight of tomato (R^2^ = 0.801), cucumber (R^2^ = 0.970), and paprika (R^2^ = 0.941). The relationship between the estimated leaf area and measured total dry weight without fruit is shown in Figure 10B. A significant correlation was observed between the estimated leaf area and measured total dry weight of tomato (R^2^ = 0.743), cucumber (R^2^ = 0.973), and paprika (R^2^ = 0.929).

**Figure 11:**
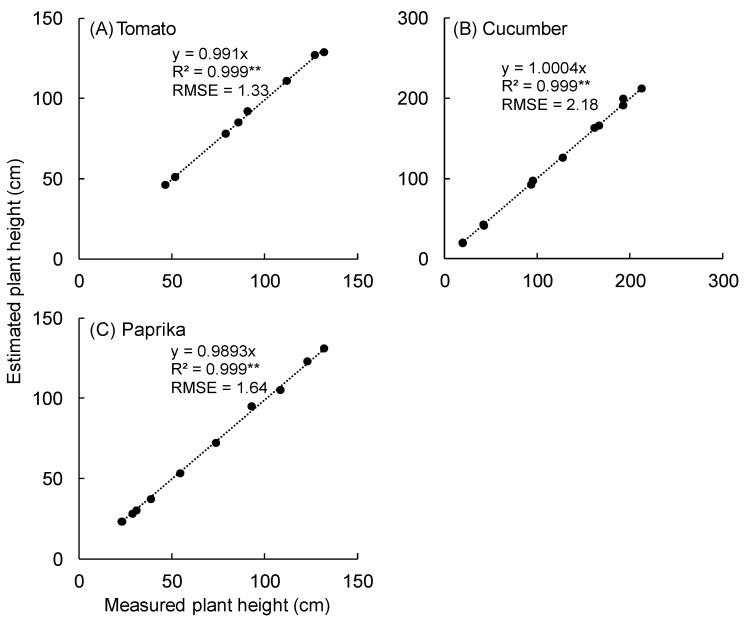
Relationship between measured plant height and estimated plant height. Plant height was estimated using the measuring tool in SketchUp 2017 (Trimble Inc., Sunnyvale, CA, USA). Tomato plants were scanned using the 3D scanner (DPI-8X, Opt Technologies Co., Ltd., Tokyo, Japan) from 5 April to 23 May 2019 (**A**). Cucumber plants were scanned from 3 April to 23 May 2019 (**B**). Paprika plants were scanned from 5 April to 2 August 2019 (**C**). ** significant at *p* < 0.01 according to the regression analysis. RMSE means root mean squared error.

The relationship between measured and estimated plant height is shown in Figure 11. A significant correlation was observed between the measured and estimated plant height of tomato (R^2^ = 0.999 and RMSE = 1.33 cm) (Figure 11A), cucumber (R^2^ = 0.999 and RMSE = 2.18 cm) (Figure 11B), and paprika (R^2^ = 0.999 and RMSE = 1.64 cm) (Figure 11C).

**Figure 12:**
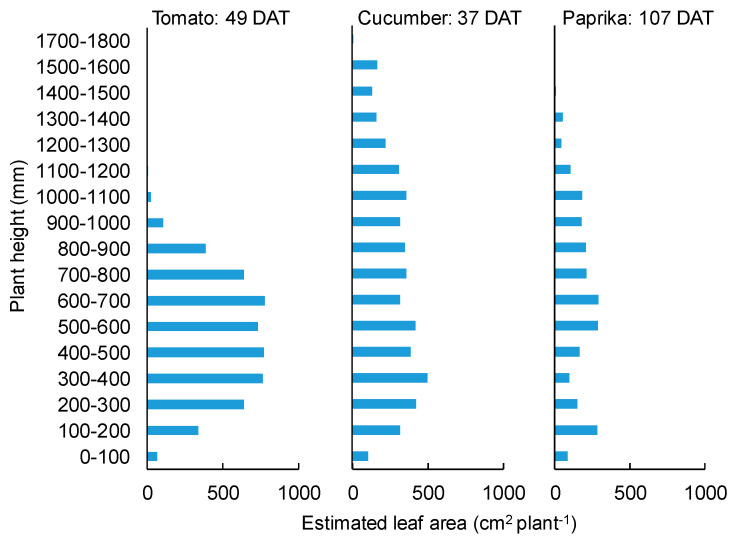
Plant structure estimated based on the height and polygon area of the canopy surface model. Tomato and cucumber canopies were scanned using the 3D scanner (DPI-8X, Opt Technologies Co., Ltd., Tokyo, Japan) on 23 May 2019. Paprika canopies were scanned on 12 June 2019.

The canopy structures of tomato (49 DAT), cucumber (37 DAT), and paprika (107 DAT) obtained by the estimated leaf area at each height are shown in Figure 12.

### 3.2. Yield

**Figure 13:**
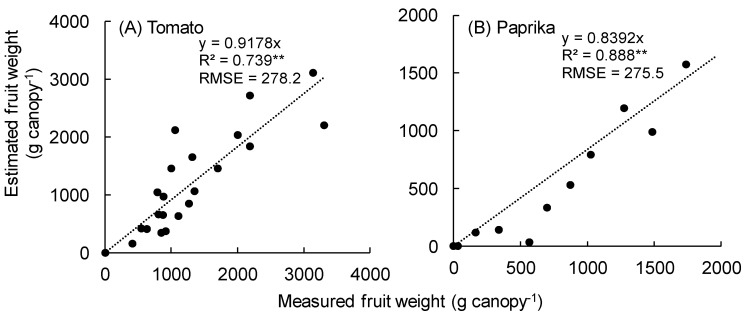
Relationship between the measured and estimated fruit weights. (**A**,**B**) show the fruit weights of tomato and paprika, respectively. Fruit weight was estimated using the volume calculated by the fruit voxel model and density. Fruit volume was calculated by Meshmixier 3.5 (Autodesk Inc., San Rafael, USA). ** significant at *p* < 0.01 according to the regression analysis. RMSE means root mean squared error.

The relationship between measured and estimated fruits weight calculated by the solid model is shown in Figure 13. A significant correlation was observed between the measured and estimated fruit weights of tomato (R^2^ = 0.739 and RMSE = 278.2 g) (Figure 13A) and paprika (R^2^ = 0.888 and RMSE = 275.5 g) (Figure 13B). Notably, the estimated fruit weight of paprika tended to be underestimated compared with the actual fruit weight.

## 4. Discussion

### 4.1. Growth

Regarding the estimation of leaf area per plant (Figure 7), the R^2^ of tomato was lower than that of cucumber and paprika. This was because tomato plants have complicated leaflets making it difficult to construct a surface model. Notably, unnecessary polygons were constructed in the tomato surface model because of noise (unnecessary point cloud; known as the drift point) between leaflets. Many denoising technologies have been developed [34]. For example, Zhou et al. (2020) eliminated the drift point using a non-interactive dual threshold denoising method [34], indicating that an accurate plant surface model could be constructed using this method. Conversely, cucumber and paprika plants could be easily scanned to enable the construction of a surface model because they have simple leaves.

Regarding the estimation of LAI (Figure 8), the R^2^ of tomato was also lower than that of cucumber and paprika, for the same reason described above. In addition, in a few surface models of tomato, the LAI was underestimated because it was difficult to acquire point cloud data inside the canopy. The LAI of paprika tended to be underestimated because the overlap of leaves in the lower layer was greater than that of other plants for morphological reasons, and point cloud data were lacking in low leaves. It was easy to estimate the LAI of cucumber because there was little overlap of leaves inside the canopy. In addition, the advantage of 3D models over 2D ones is the ability to get images from various perspectives and provides the information on the plant growth and physiological condition [11]. Therefore, when the 3D scanner was used, objects other than the target had little influence on estimates of growth parameters compared with the use of 2D images.

Ahn et al. (2015) reported a correlation between leaf fresh weight and leaf area of cucumber [35]. In our study, a significant correlation between the measured leaf area and leaf dry weight or total dry weight per plant was also observed (Figure 9). The R^2^ of the relationship between the estimated leaf area and measured leaf dry weight or total dry weight per tomato plant was lower than that of cucumber and paprika (Figure 10). This was because the leaf area of the tomato surface model was of low accuracy compared with that of other plants. Therefore, the R^2^ of cucumber and paprika was higher than that of tomato. In addition, when estimating the total dry weight of the plant using the relationship between total leaf area and dry weight, unchanged dry matter distribution to each organ is needed during cultivation. The inclination of the approximate curves for the relationship between total leaf area and total leaf dry weight or total dry weight (Figure 9) differed between tomato, cucumber, and paprika. This was because the specific leaf area and dry matter distribution changed in a species-dependent manner. Based on this, here, if the leaf area is estimated, it will be possible to estimate the total dry weight using the equation for the relationship between total leaf area and dry weight (Figure 9). Therefore, application of this approach to plant canopies to estimate the total plant dry weight will enable the optimal greenhouse environment for growing fruit vegetables to be determined.

Plant height was estimated with good accuracy (R^2^ = ~1.0), regardless of plant species (Figure 11), because the influence of leaf shape and overlap was small when plants were scanned by the 3D scanner. In addition, it was possible to estimate the canopy structure (Figure 12) because plant height and leaf area could be estimated (Figure 7 and Figure 11). Therefore, the canopy 3D model provided canopy structure information. The canopy structure changes depending on plant species and cultivation environment. Hence, the above information can be used for cultivation management such as defoliation. In addition, the estimation of canopy structure is useful to discuss the status of light environment in different leaf layers under various light conditions. The plant structure is an important factor to affect the light interception and photosynthesis of plants [36]. Kim et al. (2020) estimated the light interception on the plant surface using a 3D plant model and optical simulation and revealed the effect of the accuracy of the 3D structural model on the estimated light interception and photosynthesis [36]. It is necessary to maintain the canopy structure that increases light interception and photosynthesis by the cultivation management for improving productivity.

### 4.2. Yield

The R^2^ of the estimated paprika fruit weight was higher than that of tomato (Figure 13), because the LAI of paprika was smaller than that of tomato; therefore, fruit point cloud data were easily obtained inside the canopy. In addition, several estimated fruit weights of tomato were heavier than the actual weights (Figure 13A) because some unripe tomatoes were detected as noise.

The approximate curves used to evaluate the relationship between measured and estimated fruit weights (Figure 13) underestimated the fruit weight. This was because it was difficult to scan behind tomato and paprika fruits because of the canopy structure, resulting in missing point cloud data. Dadwal and Banga (2012) reported that when the color image segmentation was conducted to estimate the ripeness level of apple, four images of a single fruit from four different directions was prepared [31]. In addition, the RGB settings used (0–255) to detect fruit point cloud affected the output data. Therefore, the detection of noise in the point cloud, aside from fruits, will be reduced using optimal RGB settings. If objects similar to the color of the fruit exist inside the greenhouse, the false detection of fruits probably occurs. Moreover, the color temperature over the fruit changed depending on the date, time, and weather [19]. Therefore, when scanning the canopy, the use of artificial light at night to maintain a stable light environment is recommended to detect fruits. Teixidó et al. (2012) reported that the use of a color-based detection algorithm under low light intensity to detect peach resulted in the upper part of an unripe fruit being mistaken for part of a leaf [37]. Rose et al. (2016) acquired point cloud data to detect grape fruits using homogeneous lighting after sunset [38]. Font et al. (2014) counted several red grapes based on the detection of specular reflection peaks from the spherical surface of the grapes using high-resolution images taken under artificial light at night [39]. Thus, the use of artificial light at night is most effective when a 3D scanner is used to scan the canopy to acquire point cloud data and detect mature fruits. In addition, Malik et al. (2018) converted RGB images to HSV when detecting tomato fruits, because the hue component was less sensitive to variations in lighting [40]. The saturation component was not affected by light quality [19]. Therefore, converting the RGB value of point cloud data acquired by a 3D scanner to HSV or HSL will improve the accuracy of fruit detection. El-Bendary et al. (2015) evaluated tomato ripeness using machine learning with an image of fruits after converting the RGB to HSV, and obtained a classification accuracy of 90.8% [41].

Therefore, it was possible to detect fruit and to estimate their weight using the RGB value of point cloud data. In addition, this method will automatically provide growth monitoring (leaf area, LAI, plant height, and fruit weight) because it will be possible to divide the canopy point cloud data including the greenhouse structural material, and cultivation bench, into plants using color information. In the civil engineering field, several types of objects, including forest and signal, have been separated from point cloud data using color information [42,43]. An editor for point cloud data was recently developed [44], which is expected to be used in the agricultural field in the future.

## 5. Conclusions

We constructed surface models of individual plants and canopies of fruit vegetables (tomato, cucumber, and paprika) using a 3D scanner to estimate leaf area, LAI, and plant height. Using this approach, a significant correlation was observed between the measured and estimated values. The R^2^ was > 0.8, except for the LAI of tomato. In addition, a linear relationship was found between the total leaf area and total dry weight without fruit. Therefore, the dry weight of the plant can be predicted using the estimated leaf area. We were able to predict the canopy structure of each fruit vegetable by integrating the estimated leaf area at each height of the canopy surface models.

We detected tomato and paprika fruits to estimate fruit weights from the point cloud data acquired by the 3D scanner using the RGB values and constructed a solid model of fruits. The fruit weight was estimated using the volume of the solid model and the fruit density. A significant correlation (tomato: R^2^ = 0.739, paprika: R^2^ = 0.888) was observed between the measured and estimated fruit weights.

Therefore, using this method, it was possible to estimate multiple growth parameters of fruit vegetables simultaneously in a non-destructive manner. In the future, the 3D scanner is expected to be used to monitor the growth and yield of fruit vegetables.
